# Forecasting the Amount of Waste-Sewage Water Discharged into the Yangtze River Basin Based on the Optimal Fractional Order Grey Model

**DOI:** 10.3390/ijerph15010020

**Published:** 2017-12-23

**Authors:** Shuliang Li, Wei Meng, Yufeng Xie

**Affiliations:** 1 College of Business Planning, Chongqing Technology and Business University, Chongqing 400067, China; mengwei@ctbu.edu.cn; 2 Chongqing Key Laboratory of Electronic Commerce and Supply Chain System, Chongqing 400067, China; 15826050967@163.com

**Keywords:** grey theory, fractional order grey model, particle swarm optimization, the Yangtze River basin, discharge amount of waste-sewage water, simulation and prediction

## Abstract

With the rapid development of the Yangtze River economic belt, the amount of waste-sewage water discharged into the Yangtze River basin increases sharply year by year, which has impeded the sustainable development of the Yangtze River basin. The water security along the Yangtze River basin is very important for China, It is something about water security of roughly one-third of China’s population and the sustainable development of the 19 provinces, municipalities and autonomous regions among the Yangtze River basin. Therefore, a scientific prediction of the amount of waste-sewage water discharged into Yangtze River basin has a positive significance on sustainable development of industry belt along with Yangtze River basin. This paper builds the fractional DWSGM (1,1) (DWSGM (1,1) model is short for Discharge amount of Waste Sewage Grey Model for one order equation and one variable) model based on the fractional accumulating generation operator and fractional reducing operator, and calculates the optimal order of “r” by using particle swarm optimization (PSO) algorithm for solving the minimum average relative simulation error. Meanwhile, the simulation performance of DWSGM (1,1) model with the optimal fractional order is tested by comparing the simulation results of grey prediction models with different orders. Finally, the optimal fractional order DWSGM (1,1) grey model is applied to predict the amount of waste-sewage water discharged into the Yangtze River basin, and corresponding countermeasures and suggestions are put forward through analyzing and comparing the prediction results. This paper has positive significance on enriching the fractional order modeling method of the grey system.

## 1. Introduction

On 26 January 2016, General Secretary Xi Jinping stressed that the priority ideas of China should be adopted to promote the development of the Yangtze River economic belt at the Twelfth Meeting of the Central Financial Work Leading Group. This should adhere to the ecological priority and green development, and see the ecological and environmental protection as the most important thing. All the economic activities related to the Yangtze River should be based on the premise of not destroying the ecological environment [1]. In October 2017, the report of the nineteenth National People’s Congress of the Communist Party of China pointed out: “we must establish and practice the concept that clear waters and lush mountains are invaluable assets…The ecological environment should be treated as being done to our life”.

The amount of waste-sewage water discharged into the Yangtze River basin refers to the following areas: above Shigu of Jinsha river, under Shigu of Jinsha river, Mintuo river, Jialing river, Wujiang river, Yibin to Yichang, Dongting lake water system, Han river, Poyang lake water system, Yichang to Hukou, the main river below Hukou, Taihu lake water system, etc. 12 water secondary areas of industrial wastewater and domestic sewage in total. However, the amount of waste-sewage water discharged into the Yangtze River basin increased from 15 billion tons per year in the 1980s to 34.67 billion tons in 2015, with an increase of 131% and an average annual increase of more than 5%. Therefore, the scientific prediction of the amount of waste-sewage water discharged into the Yangtze River basin apparently has great practical significance in order to complete the strategy that “the water determines both the city’s development and the economic productivity” along the Yangtze River Basin of the “13th Five-Year” National Plan.

The amount of waste-sewage water discharged into the Yangtze River basin is affected by many factors such as natural, economic, social, population, development, utilization and the protection system of the upper, middle and lower reaches of the river basin. The data are characterized by uncertainty of influencing factors, small sample data and irregular distribution. The characteristics of “small data and uncertainty” increased the difficulty in predicting for the discharge amount of waste-sewage water of the Yangtze River basin by the traditional analysis and prediction model. The Grey prediction model is one of the important methods to research and solve the “small data and uncertainty” prediction problem, and how to ensure the reliability and stability of the grey prediction model with small data has attracted much attention from all walks of life [2]. After an accumulated generating operation, the non-negative smooth sequence usually shows an approximately exponential law, namely the grey exponential law. Therefore, the accumulated generating method is a common method of grey theory used to weaken the original sequence, and it is the foundation for constructing and optimizing the grey prediction model. Experiencing two important stages from the accumulation of integer order generation to the fractional accumulating generation, and this method has been used to produce many important scientific results [3]. Professor Deng Julong [4], using cumulative and reductive method as the main research methods of the grey system, presented the concept and properties of cumulating generation of the grey exponential law, made classification discussion to the exponential law of origin distribution, positive and negative distribution, and demonstrated the relationship between the smoothness of function and grey exponential law which lays a theoretical foundation for grey modelling of prediction. However, the grey prediction model based on the integer order cumulating generation method belongs to the integer order derivative model and it also belongs to the ideal memory model. Therefore, it is not suitable for describing some irregular phenomena [5]. In order to reveal the essential characteristics under behaviour of objects clearly, it is necessary to construct a more general fractional grey accumulating generation method. Xinping Xiao revealed modelling mechanism of GM (1,1) (grey model) by matrix, and studied the fractional model of GGM (1,1) (generalized grey model) and its properties [6]; Meng Wei [7,8,9,10,11], Wu Lifeng [12,13,14,15,16] had conducted systematic research on the fractional order accumulating generation method, the fractional order grey forecast model modelling method and its application question, and had obtained some important properties about the grey forecast model’s perturbation, the information priority, the robustness and so on; Zeng Bo, Liu Sifeng [17,18,19,20,21,22] had studied grey system analysis, grey method, grey models and its applications, and gotten fruitful results; Mao Shuhua [23,24] expanded the research of fractional gray prediction model, and constructed fractional order multivariable grey model GM (1,N,τ) with time-lag delayτ and FGM (q,1) (fractional order grey model) model based on FAGM (1,1) (fractional order accumulative grey model) model. The optimal accumulating generation order of fractional order is determined by particle swarm optimization and matrix analysis, and it is proved that the model has better simulation performance by comparing the actual cases; Yang Baohua [25], Wang Junfang [26] and Pan Xianjun [27] determine the optimal accumulating order or exponential respectively by using quantum genetic algorithm, regularization algorithm and genetic algorithm so as to improve the prediction accuracy of model and the new model is applied to settlement of the highway subgrade, industrial wastewater discharge rate, city water amount, weapons and equipment spare parts demand prediction etc.

The above research results indicated grey accumulating generation order extended from integer to fraction, and was of great significance to improve modelling ability and the stability of the grey prediction model. Based on the references [7], this paper gives the fractional operator GM (1,1) model on the basis of the fractional accumulating generation operator and fractional reducing operator, and worked out the optimal order of “r” by using particle swarm optimization algorithm for solving the minimum average relative error. Meanwhile, the simulation performance of the optimal fractional order grey model is tested by comparing the simulation results of grey prediction models with different order. The results show that the new model has a better simulation and prediction performance than those of the other grey prediction models, because it gives the best fractional cumulating generation operator by using particle swarm optimization algorithm for improving the performance of simulation and prediction. Finally, the optimal fractional order grey model is applied to predict the amount of waste-sewage water discharged into the Yangtze River basin, and corresponding countermeasures and suggestions are put forward through analyzing and comparing the prediction results.

The main section of this paper is organized as the following order. The Section 1 is an introduction. We construct the optimal fractional order DWSGM (1,1) model by using particle swarm optimization algorithm in Section 2. In Section 3, we give the optimization order of the DWSGM (1,1) model and compare the simulation results of grey prediction models with different order. Some suggestions is given in Section 4 and we present our conclusions in Section 5.

## 2. Building the Optimal Fractional Order Grey GM (1,1) Model

This section is about the grey prediction model of the discharge amount of waste-sewage water of the Yangtze River basin. First of all, we introduce the basic concept of the accumulating and reducing generation operator with fractional order. Then we constructed the DWSGM (1,1) model with different orders and compared simulation error and results of this model, studied the parameter estimation method, time response formula, final restore formula and test performance of the DWSGM (1,1) model with right order, in order to offer the method for predicting the discharge amount of waste-sewage water of the Yangtze River basin. First, we derive the Gamma function recursive relationships in order to express the result for the fractional accumulating generation sequence.

**Definition** **1.** *Assume* n∈R *and* n∉{0,−1,−2,−3,⋯}*,* Γ(n) *is the Gamma function for real Numbers “n”,* $t\in R^{+}$*,* *the expression formula is*

(1) $$\Gamma \left(n\right)=\int_{0}^{\infty }e^{-t}t^{n-1}dt$$

We can derive the Gamma function has the following recurrence relation, through integral method of division. That is

(2) Γ(n+1)=nΓ(n)

Specially, when n∈N, then

(3) $$\Gamma \left(n+1\right)=\int_{0}^{\infty }e^{-t}t^{n}dt=n!$$

Hence, $\Gamma \left(\frac{1}{2}\right)=\sqrt{\pi }$, Γ(1)=Γ(2)=1, Γ(3)=2!, Γ(4)=3!, …, Γ(n)=(n−1)!.

That is obvious fact, the Gamma function is a generalization of the factorial in real number field.

**Definition** **2.** *Assume that* $D_{W}^{\left(0\right)}=\left(d_{w}^{\left(0\right)}\left(1\right),d_{w}^{\left(0\right)}\left(2\right),\cdots ,d_{w}^{\left(0\right)}\left(n\right)\right)$ *is the time series data of the amount of waste-sewage water discharged into the Yangtze River basin, then* $D_{W}^{\left(1\right)}=\left(d_{w}^{\left(1\right)}\left(1\right),d_{w}^{\left(1\right)}\left(2\right),\cdots ,d_{w}^{\left(1\right)}\left(n\right)\right)$ *is called the accumulating generation sequence with one order of* $D_{W}^{\left(0\right)}$*, where*

(4) $$d_{w}^{\left(1\right)}\left(k\right)=\sum_{i=1}^{k}d_{w}^{\left(0\right)}\left(i\right),\;k=1,2,\cdots ,n$$

**Theorem** **1** [5]**.** *Assume that* $D_{W}^{\left(0\right)}=\left(d_{w}^{\left(0\right)}\left(1\right),d_{w}^{\left(0\right)}\left(2\right),\cdots ,d_{w}^{\left(0\right)}\left(n\right)\right)$ *is the original sequence,* $r=R^{+}$*,* $D_{W}^{\left(r\right)}=\left(d_{w}^{\left(r\right)}\left(1\right),d_{w}^{\left(r\right)}\left(2\right),\cdots ,d_{w}^{\left(r\right)}\left(n\right)\right)$ *is called the accumulating generation sequence with order “r” of* $D_{W}^{\left(0\right)}$*, where*

(5) $$d_{w}^{\left(r\right)}\left(k\right)=\sum_{i=1}^{k}\frac{\Gamma \left(r+k-i\right)}{\Gamma \left(k-i+1\right)\Gamma \left(r\right)}d_{w}^{\left(0\right)}\left(i\right),\;k=1,2,\cdots ,n$$

The expression of matrix of Formula (5) can be expressed as follows:

(6) $$d_{w}^{\left(r\right)}\left(k\right)=\left(\begin{array}{cccc} \frac{\Gamma \left(r+k-1\right)}{\Gamma \left(k\right)\Gamma \left(r\right)} & \frac{\Gamma \left(r+k-2\right)}{\Gamma \left(k-1\right)\Gamma \left(r\right)} & \cdots & \frac{\Gamma \left(r\right)}{\Gamma \left(1\right)\Gamma \left(r\right)} \end{array}\right)\cdot \left(\begin{array}{c} d_{w}^{\left(0\right)}\left(1\right)\\ d_{w}^{\left(0\right)}\left(2\right)\\ \cdots \\ d_{w}^{\left(0\right)}\left(k\right) \end{array}\right),\;k=1,2,\cdots ,n$$

**Definition** **3** [3]**.** *Assume that* $D_{W}^{\left(0\right)}=\left(d_{w}^{\left(0\right)}\left(1\right),d_{w}^{\left(0\right)}\left(2\right),\cdots ,d_{w}^{\left(0\right)}\left(n\right)\right)$ *is the original sequence,* $D_{W}^{\left(-1\right)}=\left(d_{w}^{\left(-1\right)}\left(1\right),d_{w}^{\left(-1\right)}\left(1\right),\cdots ,d_{w}^{\left(-1\right)}\left(n\right)\right)$ *is called the reducing generation sequence with one order of* $D_{W}^{\left(0\right)}$*, where*

(7) $$d_{w}^{\left(-1\right)}\left(k\right)=d_{w}^{\left(0\right)}\left(k\right)-d_{w}^{\left(0\right)}\left(k-1\right),\;k=1,2,\cdots ,n$$

**Theorem** **2** [5]**.** *Assume that* $D_{W}^{\left(0\right)}=\left(d_{w}^{\left(0\right)}\left(1\right),d_{w}^{\left(0\right)}\left(2\right),\cdots ,d_{w}^{\left(0\right)}\left(n\right)\right)$ *is the original sequence,* $r\in R^{+}$*,* $D_{W}^{\left(-r\right)}=\left(d_{w}^{\left(-r\right)}\left(1\right),d_{w}^{\left(-r\right)}\left(2\right),\cdots ,d_{w}^{\left(-r\right)}\left(n\right)\right)$ *is called the reducing generation sequence with order “r” of* $D_{W}^{\left(0\right)}$ *, where*

(8) $$d_{w}^{\left(-r\right)}\left(k\right)=\sum_{i=0}^{k-1}{\left(-1\right)}^{i}\frac{\Gamma \left(r+1\right)}{\Gamma \left(i+1\right)\Gamma \left(r-i+1\right)}d_{w}^{\left(0\right)}\left(k-i\right),\;k=1,2,\cdots ,n$$

The expression of matrix of Formula (8) can be expressed as follows:

(9) $$d_{w}^{\left(-r\right)}\left(k\right)=\left(\begin{array}{cccc} \frac{\Gamma \left(r+1\right)}{\Gamma \left(1\right)\Gamma \left(r+1\right)} & -\frac{\Gamma \left(r+1\right)}{\Gamma \left(2\right)\Gamma \left(r\right)} & \cdots & {\left(-1\right)}^{k-1}\frac{\Gamma \left(r+1\right)}{\Gamma \left(k\right)\Gamma \left(r-k+2\right)} \end{array}\right)\cdot \left(\begin{array}{c} d_{w}^{\left(0\right)}\left(k\right)\\ d_{w}^{\left(0\right)}\left(k-1\right)\\ \cdots \\ d_{w}^{\left(0\right)}\left(r-k+1\right) \end{array}\right),\;k=1,2,\cdots ,n$$

**Theorem** **3** [5]**.** *Assume that* $D_{W}^{\left(0\right)}=\left(d_{w}^{\left(0\right)}\left(1\right),d_{w}^{\left(0\right)}\left(2\right),\cdots ,d_{w}^{\left(0\right)}\left(n\right)\right)$ *is the original sequence,* $r\in R^{+}$*,* $D_{W}^{\left(r\right)}$ *is called the accumulating generation sequence with order “r” of* $D_{W}^{\left(0\right)}$*,* $D_{W}^{\left(-r\right)}$ *is called the reducing generation sequence with order “r” of* $D_{W}^{\left(0\right)}$*, the accumulating generation operator and the reducing generation operator with order “r” are inverse operation, that is*

(10) $$D_{W}^{\left(0\right)}={\left(D_{W}^{\left(r\right)}\right)}^{\left(-r\right)}={\left(D_{W}^{\left(-r\right)}\right)}^{\left(r\right)}$$

**Definition** **4**. *Assume* $D_{W}^{\left(0\right)}$ *is stated as Definition 2,* $D_{W}^{\left(r\right)}$ *is stated as Theorem 1,* $Z^{\left(r\right)}=\left(z^{\left(r\right)}\left(2\right),z^{\left(r\right)}\left(3\right),\cdots ,z^{\left(r\right)}\left(n\right)\right)$*, where,*

$$z^{\left(r\right)}\left(k\right)=\frac{d_{w}^{\left(r\right)}\left(k\right)+d_{w}^{\left(r\right)}\left(k-1\right)}{2},\;k=2,3,\cdots ,n$$

That is

(11) $$d_{w}^{\left(r-1\right)}\left(k\right)+az^{\left(r\right)}\left(k\right)=b$$

Formula (11) is the GM (1,1) model for fractional order operator.

In particular when “r” = 1, $d_{w}^{\left(r-1\right)}\left(k\right)+az^{\left(r\right)}\left(k\right)=b$ can be changed to $d_{w}^{\left(0\right)}\left(k\right)+az^{\left(1\right)}\left(k\right)=b$, that is the even GM (1,1) model.

**Theorem** **4** [5]**.** *Assume* $D_{W}^{\left(0\right)}=\left(d_{w}^{\left(0\right)}\left(1\right),d_{w}^{\left(0\right)}\left(2\right),\cdots ,d_{w}^{\left(0\right)}\left(n\right)\right)$ *is the original sequence,* $r\in R^{+}$*,* $D_{W}^{\left(r\right)}$ *is stated as Theorem 1,* $D_{W}^{\left(-r\right)}$ *is stated as Theorem 2,* $Z^{\left(r\right)}$ *is stated as Definition 4;*

The parameter vector $\hat{a}={\left[a,b\right]}^{T}$ in $d_{w}^{\left(r-1\right)}\left(k\right)+az^{\left(r\right)}\left(k\right)=b$ of GM (1,1) model for fractional order operator can be estimated by using the ordinary least-squares (OLS),

(12) $$\hat{a}={\left(B^{T}B\right)}^{-1}B^{T}Y$$

where, Y, B can be definite as:

(13) $$Y=\left[\begin{array}{c} d_{w}^{\left(r-1\right)}\left(2\right)\\ d_{w}^{\left(r-1\right)}\left(3\right)\\ \vdots \\ d_{w}^{\left(r-1\right)}\left(n\right) \end{array}\right],\;B=\left[\begin{array}{cc} -z^{\left(r\right)}\left(2\right) & 1\\ -z^{\left(r\right)}\left(3\right) & 1\\ \vdots & \vdots \\ -z^{\left(r\right)}\left(n\right) & 1 \end{array}\right]$$

Since,

$$d_{w}^{\left(r-1\right)}\left(k\right)=d_{w}^{\left(r\right)}\left(k\right)-d_{w}^{\left(r\right)}\left(k-1\right)$$

$$=\sum_{i=1}^{k}\frac{\Gamma \left(r+k-i\right)}{\Gamma \left(k-i+1\right)\Gamma \left(r\right)}d_{w}^{\left(0\right)}\left(i\right)-\sum_{i=1}^{k-1}\frac{\Gamma \left(r+k-i-1\right)}{\Gamma \left(k-i\right)\Gamma \left(r\right)}d_{w}^{\left(0\right)}\left(i\right),\;k=2,3,\cdots ,n$$

Then

$$z^{\left(r\right)}\left(k\right)=\frac{\sum_{i=1}^{k}\frac{\Gamma \left(r+k-i\right)}{\Gamma \left(k-i+1\right)\Gamma \left(r\right)}d_{w}^{\left(0\right)}\left(i\right)+\sum_{i=1}^{k-1}\frac{\Gamma \left(r+k-i\right)}{\Gamma \left(k-i+1\right)\Gamma \left(r\right)}d_{w}^{\left(0\right)}\left(i\right)}{2}$$

$$Y=\left[\begin{array}{c} \sum_{i=1}^{2}\frac{\Gamma \left(r+2-i\right)}{\Gamma \left(2-i+1\right)\Gamma \left(r\right)}d_{w}^{\left(0\right)}\left(i\right)-\sum_{i=1}^{1}\frac{\Gamma \left(r+2-1-i\right)}{\Gamma \left(2-i\right)\Gamma \left(r\right)}d_{w}^{\left(0\right)}\left(i\right)\\ \sum_{i=1}^{3}\frac{\Gamma \left(r+3-i\right)}{\Gamma \left(3-i+1\right)\Gamma \left(r\right)}d_{w}^{\left(0\right)}\left(i\right)-\sum_{i=1}^{2}\frac{\Gamma \left(r+3-1-i\right)}{\Gamma \left(3-i\right)\Gamma \left(r\right)}d_{w}^{\left(0\right)}\left(i\right)\\ \vdots \\ \sum_{i=1}^{n}\frac{\Gamma \left(r+n-i\right)}{\Gamma \left(n-i+1\right)\Gamma \left(r\right)}d_{w}^{\left(0\right)}\left(i\right)-\sum_{i=1}^{n-1}\frac{\Gamma \left(r+n-1-i\right)}{\Gamma \left(n-i\right)\Gamma \left(r\right)}d_{w}^{\left(0\right)}\left(i\right) \end{array}\right]=\left[\begin{array}{c} \left(r-1\right)d_{w}^{\left(0\right)}\left(1\right)+d_{w}^{\left(0\right)}\left(2\right)\\ \frac{r\left(r-1\right)}{2}d_{w}^{\left(0\right)}\left(1\right)+\left(r-1\right)d_{w}^{\left(0\right)}\left(2\right)+d_{w}^{\left(0\right)}\left(3\right)+\\ \vdots \\ \sum_{i=1}^{n}\frac{\Gamma \left(r+n-i\right)}{\Gamma \left(n-i+1\right)\Gamma \left(r\right)}d_{w}^{\left(0\right)}\left(i\right)-\sum_{i=1}^{n-1}\frac{\Gamma \left(r+n-1-i\right)}{\Gamma \left(n-i\right)\Gamma \left(r\right)}d_{w}^{\left(0\right)}\left(i\right) \end{array}\right]$$

$$B=\left[\begin{array}{cc} -\frac{d_{w}^{\left(r\right)}\left(1\right)+d_{w}^{\left(r\right)}\left(2\right)}{2} & 1\\ -\frac{d_{w}^{\left(r\right)}\left(2\right)+d_{w}^{\left(r\right)}\left(3\right)}{2} & 1\\ \vdots & \vdots \\ -\frac{d_{w}^{\left(r\right)}\left(n-1\right)+d_{w}^{\left(r\right)}\left(n\right)}{2} & 1 \end{array}\right]=\left[\begin{array}{cc} -\frac{1}{2}\left[\sum_{i=1}^{2}\frac{\Gamma \left(r+2-i\right)}{\Gamma \left(2-i+1\right)\Gamma \left(r\right)}d_{w}^{\left(0\right)}\left(i\right)+\sum_{i=1}^{1}\frac{\Gamma \left(r+1-i\right)}{\Gamma \left(1-i+1\right)\Gamma \left(r\right)}d_{w}^{\left(0\right)}\left(i\right)\right] & 1\\ -\frac{1}{2}\left[\sum_{i=1}^{3}\frac{\Gamma \left(r+3-i\right)}{\Gamma \left(3-i+1\right)\Gamma \left(r\right)}d_{w}^{\left(0\right)}\left(i\right)+\sum_{i=1}^{2}\frac{\Gamma \left(r+2-i\right)}{\Gamma \left(2-i+1\right)\Gamma \left(r\right)}d_{w}^{\left(0\right)}\left(i\right)\right] & 1\\ \vdots & \vdots \\ -\frac{1}{2}\left[\sum_{i=1}^{n}\frac{\Gamma \left(r+n-i\right)}{\Gamma \left(n-i+1\right)\Gamma \left(r\right)}d_{w}^{\left(0\right)}\left(i\right)+\sum_{i=1}^{n-1}\frac{\Gamma \left(r+n-1-i\right)}{\Gamma \left(n-1-i+1\right)\Gamma \left(r\right)}d_{w}^{\left(0\right)}\left(i\right)\right] & 1 \end{array}\right]$$

$$=\left[\begin{array}{cc} -\frac{1}{2}\left[\left(r+1\right)d_{w}^{\left(0\right)}\left(1\right)+d_{w}^{\left(0\right)}\left(2\right)\right] & 1\\ -\frac{1}{2}\left[\frac{r\left(r+3\right)}{2}d_{w}^{\left(0\right)}\left(1\right)+\left(r+1\right)d_{w}^{\left(0\right)}\left(2\right)+d_{w}^{\left(0\right)}\left(3\right)\right] & 1\\ \vdots & \vdots \\ -\frac{1}{2}\left[\sum_{i=1}^{n}\frac{\Gamma \left(r+n-i\right)}{\Gamma \left(n-i+1\right)\Gamma \left(r\right)}d_{w}^{\left(0\right)}\left(i\right)+\sum_{i=1}^{n-1}\frac{\Gamma \left(r+n-i\right)}{\Gamma \left(n-i+1\right)\Gamma \left(r\right)}d_{w}^{\left(0\right)}\left(i\right)\right] & 1 \end{array}\right]$$

**Definition** **5.** *Set*

(14) $$\frac{d\;d_{w}^{\left(r\right)}}{dt}+ad_{w}^{\left(r\right)}=b$$

*As the whitenization differential equation for* $d_{w}^{\left(r-1\right)}\left(k\right)+az^{\left(r\right)}\left(k\right)=b$ *of GM (1,1) model for fractional order operator.*

**Theorem** **5** [5]**.** *Assume* B*,* Y*,* $\hat{a}$ *is stated as Theorem 4,* $\hat{a}={\left[a,b\right]}^{T}={\left(B^{T}B\right)}^{-1}B^{T}Y$*, then*

(1) The answer for the whitenization differential equation $\frac{dd_{w}^{\left(r\right)}}{dt}+ad_{w}^{\left(r\right)}=b$ of GM (1,1) model for fractional order operator, that is the time response function as follows:
  (15) $${\hat{d}}_{w}^{\left(r\right)}\left(t\right)=\left(d_{w}^{\left(r\right)}\left(1\right)-\frac{b}{a}\right)e^{-at}+\frac{b}{a}$$
(2) The time response sequence for $d_{w}^{\left(r-1\right)}\left(k\right)+az^{\left(r\right)}\left(k\right)=b$ of GM (1,1) model for fractional order operator is as follows:
  (16) $${\hat{d}}_{w}^{\left(r\right)}\left(k\right)=\left(d_{w}^{\left(0\right)}\left(1\right)-\frac{b}{a}\right)e^{-a\left(k-1\right)}+\frac{b}{a},\;k=2,3,\cdots ,n$$
(3) The reduced value is as follows:
  (17) $${\hat{d}}_{w}^{\left(0\right)}\left(k\right)={\left({\hat{d}}_{w}^{\left(r\right)}\right)}^{\left(-r\right)}\left(k\right)=\sum_{i=0}^{k-1}{\left(-1\right)}^{i}\frac{\Gamma \left(r+1\right)}{\Gamma \left(i+1\right)\Gamma \left(r-i+1\right)}{\hat{d}}_{w}^{\left(r\right)}\left(k-i\right),\;k=2,3,\cdots ,n$$

Special case

$${\hat{d}}_{w}^{\left(0\right)}\left(1\right)=d_{w}^{\left(0\right)}\left(1\right)$$

Formula (17) is called the Discharge amount of Waste Sewage Grey Model for one order equation and one variable, which is written as DWSGM (1,1) model for short. We do not know the order “r” in this model and we can calculate the optimization order of the DWSGM (1,1) model by using the Particle Swarm Optimization (PSO).

## 3. The Prediction for the Amount of Waste-Sewage Water Discharged into the Yangtze River Basin and Results Comparison

### 3.1. The Optimization Order of the DWSGM (1,1) Model

According to the previous analysis, we know that the order number is an important parameter influencing the performances of the grey prediction model. The same grey prediction model with different order number usually has different model accuracy, so how to choose a relatively optimal order has a great significance for improving the prediction accuracy of the discharge amount of waste-sewage water of the Yangtze River basin. Therefore, The Particle Swarm Optimization (PSO) will be used for optimizing the order of the DWSGM (1,1) model in this article. The Particle Swarm Optimization (PSO) proposed by Eberhart and Kennedy in 1995 is a global optimization evolutionary algorithm. In this paper, we will use the PSO to optimize the order of the DWSGM (1,1) model, and the optimization order of the DWSGM (1,1) model is sought under the condition of the least mean relative simulative errors, as follows.

(18) $$\min f\left(r\right)=\frac{1}{n-1}\sum_{k=2}^{n}\frac{\left|{\hat{d}}_{w}^{\left(0\right)}\left(k\right)-d_{w}^{\left(0\right)}\left(k\right)\right|}{d_{w}^{\left(0\right)}\left(k\right)},r\in R^{+}$$

The major steps of the optimization order for the DWSGM (1,1) model are as follows.

Step 1: Initializing randomly the position (pBest) and speed for each particle, when pBest=1, it is just the traditional GM (1,1) model.

Step 2: Setting pBest as the current position and gBest as the optimal particle position in initial swarm.

Step 3: Computing the mean relative simulative percentage errors (MRSPE) of the proposed DWSGM (1,1) model when r=pBest.

Step 4: Executing the following calculations for all particles.

(a) Updating the position and velocity of particle.
  (19) $$V=\omega \times V+c_{1}\times rand\times \left(p\mathrm{Best}-\mathrm{Present}\right)+c_{2}\times rand\times \left(g\mathrm{Best}-\mathrm{Present}\right)$$
  (20) $$\omega =\omega_{\max}-\frac{\mathrm{run}\left(\omega_{\max}-\omega_{\min}\right)}{\mathrm{run}\mathrm{Max}}$$
  (21) Present=Present+V
(b) If the fitness of this particle is superior to pBest, the new position is set to pBest.
(c) If the fitness of this particle is superior to gBest, the new position is set to gBest.

Step 5: Computing the variance $\sigma^{2}$ of the group fitness and f(gBest).

(22) $$\sigma^{2}={\sum_{i=1}^{n}\left(\frac{f_{i}-f_{avg}}{f}\right)}^{2}$$

(23) $$f=\left\{ \begin{array}{l} \max\left\{\left|f_{i}-f_{avg}\right|\right\},\left\{\left|f_{i}-f_{avg}\right|\right\}>1\\ 1,\mathrm{others} \end{array}\right.$$

Step 6: Computing the probability of variation $p_{m}$.

(24) $$p_{m}=\left\{ \begin{array}{l} k,\sigma^{2}<\sigma_{d}^{2}\;\mathrm{and}\;f\left(g\mathrm{Best}\right)>f_{d}\;\\ 0,\mathrm{others} \end{array}\right.$$

Step 7: Generating random numbers ε∈[0,1], if $\varepsilon <p_{m}$, executing the variation operation according Equation (23), else go to Step 8.

(25) $$g{\mathrm{Best}}_{k}=g{\mathrm{Best}}_{k}\times \left(1+0.5\eta \right)$$

Step 8: Judging whether the algorithm meets the convergence rule; if it meets it, then go to Step 9, else go to Step 3.

Step 9: Outputting gBest, which is the optimal value of the order r; Outputting the simulated or forecasted values of the DWSGM (1,1) model when r=gBest.

### 3.2. The Predicted Results of the DWSGM (1,1) Model with Different Order

From Table 1, the raw sequence $D_{W}^{\left(0\right)}$ is as follows,

$$\begin{array}{ll} D_{W}^{\left(0\right)} & =\left(d_{w}^{\left(0\right)}\left(1\right),d_{w}^{\left(0\right)}\left(2\right),d_{w}^{\left(0\right)}\left(3\right),d_{w}^{\left(0\right)}\left(4\right),d_{w}^{\left(0\right)}\left(5\right),d_{w}^{\left(0\right)}\left(6\right),d_{w}^{\left(0\right)}\left(7\right),d_{w}^{\left(0\right)}\left(8\right)\right)\\ & =\left(325.11,333.15,339.0,342.1,347.4,336.7,338.8,346.7\right) \end{array}$$

In order to compare prediction performance of the DWSGM (1,1) model with different orders, We can set the order “r” for: 0.10; 0.25; 0.40; 0.55; 0.70; 0.85; 1.00; and use the PSO to optimize the best order of the DWSGM (1,1) model. That is “r” = 0.94. Calculating the parameters and simulation error of the DWSGM (1,1) model. The parameters of the DWSGM (1,1) model with different orders are as shown in Table 2, the final simulated values and errors of the DWSGM (1,1) model with different orders are as shown in Table 3, as follows.

The mathematical meanings of symbols ${\hat{d}}_{w}^{\left(0\right)}\left(k\right)$, ε(k), $\Delta_{S}\left(k\right)$ and $\bar{\Delta }$ in Table 3 are as follows:

- ${\hat{d}}_{w}^{\left(0\right)}\left(k\right)$ is the simulation data of the raw data $d_{w}^{\left(0\right)}\left(k\right)$.
- ε(k) is the Residual Error’s absolute value of ${\hat{d}}_{w}^{\left(0\right)}\left(k\right)$ (RE): $\varepsilon \left(k\right)=\left|{\hat{d}}_{w}^{\left(0\right)}\left(k\right)-d_{w}^{\left(0\right)}\left(k\right)\right|$.
- $\Delta_{S}\left(k\right)$ is the Relative Simulation Percentage Error of ${\hat{d}}_{w}^{\left(0\right)}\left(k\right)$ (RSPE): $\Delta \left(k\right)=\varepsilon \left(k\right)/d_{w}^{\left(0\right)}\left(k\right)\times 100\%$.
- $\bar{\Delta }$ is the Mean Relative Percentage Error (MRPE): $\bar{\Delta }=\left(\Delta \left(2\right)+\Delta \left(3\right)+\cdots +\Delta \left(n\right)\right)/\left(n-1\right)$.

**Figure 1:**
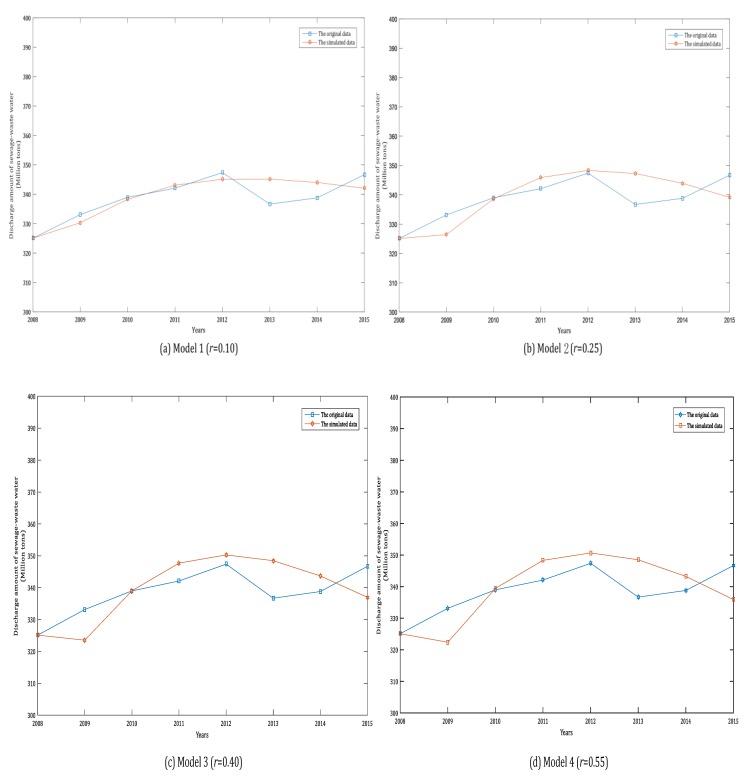

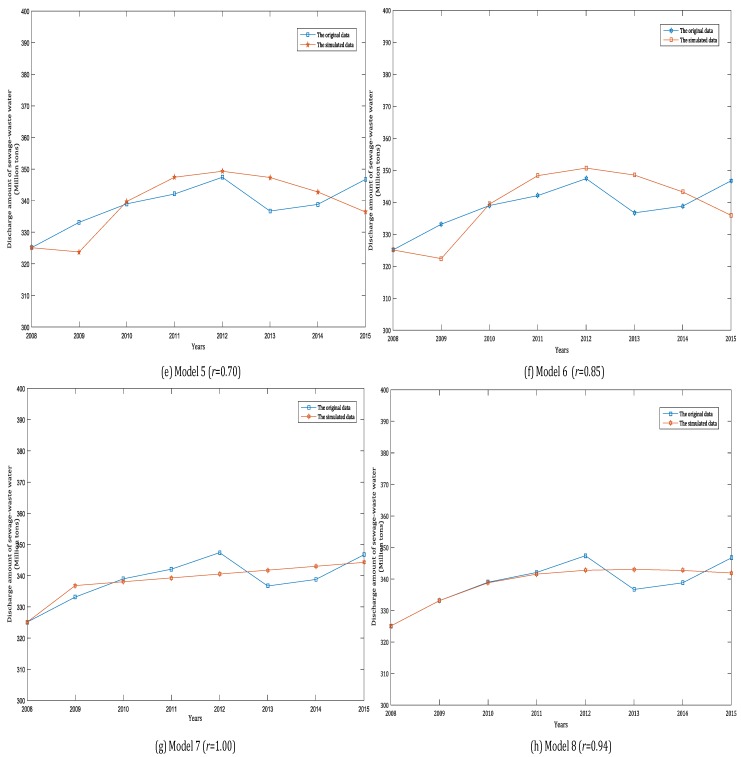
Simulated curves of the DWSGM (1,1) model with different orders.

It shows that the MRPE of the DWSGM (1,1) model with the optimal order (r = 0.94) is better than that of the rest of DWSGM (1,1) model with orders r = 0.10, 0.25, 0.40, 0.55, 0.70, 0.85 and 1.00. In order to compare the performance of the eight models clearly, we can draw the simulation curves of the above eight models based on the data in Table 2 by MATLAB (2016b, MathWorks, MA, USA), as shown in Figure 1a–h, as follows.

**Figure 2:**
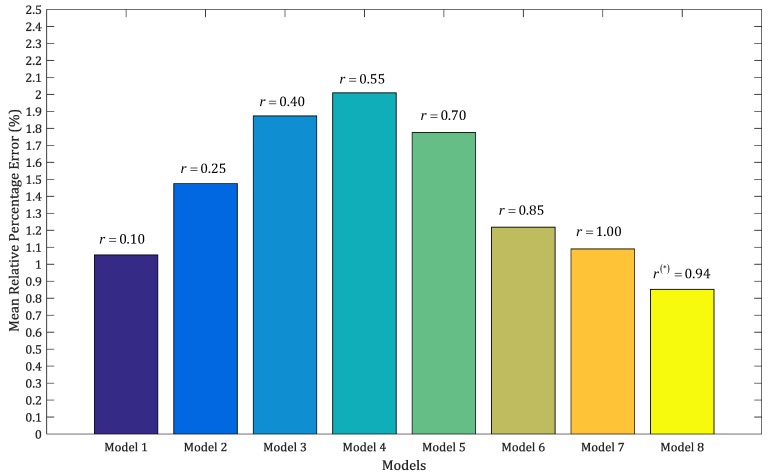
MRPEs of the DWSGM (1,1) model with different orders. MRPE: Mean Relative Percentage Error. Yellow: Model 8, r = 0.94, the optimal order; dark yellow: Model 7, r = 1; khaki: Model 6, r = 0.85; green: Model 5, r = 0.70; light blue: Model 4, r = 0.55; blue: Model 3, r = 0.40; dark blue: Model 2, r = 0.25; ink blue: Model 1, r = 0.10. The superscript symbol “*” stands for the optimal order.

According to Figure 1 and Figure 2, the performances of simulation of the DWSGM (1,1) model with the optimal order (r = 0.94) is the best among the above eight models. By checking the grey model error level reference table, we can see that the comprehensive grade of the DWSGM (1,1) model is I, which can be used for prediction.

### 3.3. The Prediction of the DWSGM (1,1)

According to the MATLAB, the final DWSGM (1,1) model is as follows,

(26) $${\hat{d}}_{w}^{\left(r\right)}\left(k\right)=\left(325.11-\frac{320.133}{0.011}\right)e^{-a\left(k-1\right)}+\frac{320.133}{0.011}$$

According to the Equation (26), the amount of waste-sewage water discharged into Yangtze River basin in the next 2018–2024 years can be predicted, as shown in Table 4.

From Table 4, we can see that the amount of waste-sewage water discharged into Yangtze River basin will reduce year by year during 2018–2024, but the total discharge amount of waste-sewage water of Yangtze River basin is still too large. In order to protect the water security of the Yangtze River basin and realize the sustainable development of China’s economy, some suggestions are put forward in the next section.

## 4. Suggestions

There are several reasons for reducing of the amount of waste-sewage water discharged into Yangtze River basin year by year during 2018–2024. First of all, China implements the most stringent protection and detection system for water resources along the Yangtze River basin from Xi Jinping Presidency. The nation must set up and practice the concept of “green water and castle peak are mountains of gold and silver”: all human activities should extend their consideration to the other ecological environment. Secondly, China has entered the stage of connotative development and no longer pursuing economic growth blindly. The government pays more attention to people’s happiness index and the protection of the ecological environment, especially the water resources protection along the Yangtze River basin. Thirdly, the government optimizes the industrial layout of high energy consumption and high pollution and does not make big developments in the Yangtze River basin.

However, we should clearly recognize that the total amount of waste-sewage water discharged into Yangtze River basin is still too large. Some suggestions are put forward as following: Firstly, the government should adjust and optimize the manufacturing industry structure along the Yangtze River basin. The excessive growth of industries that would pollute water heavily must be controlled. We should improve some effective policies and measures to optimize industrial layout along the Yangtze River basin according to the high standard of water security. At the same time, the government should limit the amount of water wasted, improve the efficiency of water consumption, and expand water function areas, as the development and utilization of total binding forces and formulate policies and measures should be passed in order to promote the development of high-tech water treatment industries along the Yangtze River basin. Secondly, it should strictly implement the water function area management system, and the dual control of restricting main pollutants into the river and raising total emissions standards (concentration)should be exercised, and translate the water pollution prevention plan into action. The government should strengthen the management of sewage outlets into the Yangtze River basin, examine the establishment of sewage outlets into the river strictly, and implement real-time monitoring and supervision and inspection of the outlets into the river. Thirdly, we will strengthen the protection of drinking water sources, implement the drinking water security standard construction of important water source area, in order to ensure the safety of drinking water in both the urban and rural Yangtze River basin. Fourthly, it should strengthen the protection of key areas, strictly control the main urban pollution prevention along the Yangtze River basin such as Shanghai, Nanjing, Wuhan, Chongqing, Panzhihua, optimize the layout of water intakes and strengthen the treatment of sewerage.

## 5. Conclusions

The water security along the Yangtze River basin is very important for China. It is something about water security of roughly one-third of China’s population and the sustainable development of the 19 provinces, municipalities and autonomous regions among the Yangtze River basin. Hence, a scientific prediction of the amount of waste-sewage water discharged into the Yangtze River basin has a positive significance on sustainable development of the industry belt along with the Yangtze River basin. For this purpose, we studied the fractional operator modelling method of the GM (1,1) model based on a fractional order accumulating generation operator and fractional reducing generation operator, and solved the minimum average relative error of the optimal order number “r” by using the particle swarm optimization algorithm. Then we constructed the DWSGM (1,1) model with different orders and compared simulation error and results of this model, studied the parameter estimation method, time response formula and performance test of the DWSGM (1,1) model with right order. Finally, the DWSGM (1,1) model was applied to forecast the discharge amount of waste-sewage water of Yangtze River basin during 2018–2024, and the suggestions were put forward according to the prediction results.

## Figures and Tables

**Table 1:** Discharge amount of waste-sewage water (DWS) of Yangtze River basin during 2008–2015.

Year	2008	2009	2010	2011	2012	2013	2014	2015
DWS	325.11	333.15	339.0	342.1	347.4	336.7	338.8	346.7

Unit: (100 Million tons).

**Table 2:** The parameters of the DWSGM (1,1) model with different order.

Order	r = 0.10		r = 0.25		r = 0.40		r = 0.55	
Parameter	a = 0.360	b = 161.910	a = 0.265	b = 180.320	a = 0.193	b = 204.126	a = 0.133	b = 231.425
Order	r = 0.70		r = 0.85		r = 1.00		r = 0.94	
Parameter	a = 0.082	b = 262.159	a = 0.037	b = 296.580	a = –0.004	b = 335.039	a = 0.011	b = 320.133

DWSGM: Discharge amount of Waste Sewage Grey Model.

**Table 3:** Simulated/forecasted values and errors of the DWSGM (1,1) model with different order.

**Year**	**Raw data** $d_{w}^{\left(0\right)}\left(k\right)$	**Model 1** **DWSGM (1,1), r = 0.10**			**Model 2** **DWSGM (1,1), r = 0.25**			**Model 3** **DWSGM (1,1), r = 0.40**			**Model 4** **DWSGM (1,1), r = 0.55**		
		${\hat{d}}_{w}^{\left(0\right)}\left(k\right)$	ε(k)	$\Delta_{S}\left(k\right)$	${\hat{d}}_{w}^{\left(0\right)}\left(k\right)$	ε(k)	$\Delta_{S}\left(k\right)$	${\hat{d}}_{w}^{\left(0\right)}\left(k\right)$	ε(k)	$\Delta_{S}\left(k\right)$	${\hat{d}}_{w}^{\left(0\right)}\left(k\right)$	ε(k)	$\Delta_{S}\left(k\right)$
2008	325.11	325.11	0.000	0.000%	325.11	0.000	0.000%	325.11	0.000	0.000%	325.11	0.000	0.000%
2009	333.15	330.368	2.782	0.835%	326.434	6.716	2.016%	323.532	9.618	2.887%	322.405	10.745	3.225%
2010	339.0	338.319	0.681	0.201%	338.653	0.347	0.102%	339.018	–0.018	0.005%	339.45	–0.45	0.133%
2011	342.1	343.114	–1.014	0.296%	345.876	–3.776	1.104%	347.716	–5.616	1.642%	348.342	–6.242	1.825%
2012	347.4	345.131	2.269	0.653%	348.31	–0.91	0.262%	350.288	–2.888	0.831%	350.699	–3.299	0.95%
2013	336.7	345.209	–8.509	2.527%	347.278	–10.58	3.142%	348.474	–11.774	3.497%	348.518	–11.818	3.51%
2014	338.8	344.03	–5.23	1.544%	343.932	–5.132	1.515%	343.691	–4.891	1.444%	343.255	–4.455	1.315%
2015	346.7	342.091	4.609	1.329%	339.125	7.575	2.185%	336.981	9.719	2.803%	335.935	10.765	3.105%
MRPE ($\bar{\Delta }$)		1.055			1.475			1.873			2.009		
Year	Raw data $d_{w}^{\left(0\right)}\left(k\right)$	Model 5 DWSGM (1,1), r = 0.70			Model 6 DWSGM (1,1), r = 0.85			Model 7 DWSGM (1,1), r = 1.00			Model 8 DWSGM (1,1), r = 0.94		
		${\hat{d}}_{w}^{\left(0\right)}\left(k\right)$	ε(k)	$\Delta_{S}\left(k\right)$	${\hat{d}}_{w}^{\left(0\right)}\left(k\right)$	ε(k)	$\Delta_{S}\left(k\right)$	${\hat{d}}_{w}^{\left(0\right)}\left(k\right)$	ε(k)	$\Delta_{S}\left(k\right)$	${\hat{d}}_{w}^{\left(0\right)}\left(k\right)$	ε(k)	$\Delta_{S}\left(k\right)$
2008	325.11	325.11	0.000	0.000%	325.11	0.000	0.000%	325.11	0.000	0.000%	325.11	0.000	0.000%
2009	333.15	323.772	9.378	2.815%	328.343	4.807	1.443%	336.835	–3.685	1.106%	333.15	0.000	0.000%
2010	339.0	339.762	–0.762	0.225%	339.53	–0.53	0.156%	338.064	0.936	0.276%	338.817	0.183	0.054%
2011	342.1	347.41	–5.31	1.552%	344.53	–2.43	0.71%	339.296	2.804	0.82%	341.566	0.534	0.156%
2012	347.4	349.291	–1.891	0.544%	345.911	1.489	0.429%	340.534	6.866	1.976%	342.785	4.615	1.328%
2013	336.7	347.329	–10.63	3.157%	344.995	–8.295	2.464%	341.776	–5.076	1.508%	343.071	–6.371	1.892%
2014	338.8	342.766	–3.966	1.171%	342.531	–3.731	1.101%	343.021	–4.221	1.246%	342.728	–3.928	1.159%
2015	346.7	336.423	10.28	2.964%	338.982	7.718	2.226%	344.273	2.427	0.700%	341.937	4.763	1.374%
MRPE ($\bar{\Delta }$)		1.775			1.218			1.090			0.852		

MRPE: Mean relative percentage error.

**Table 4:** Prediction data of the discharge amount of waste-sewage water (DWS) into Yangtze River basin during 2018–2024.

Year	2018	2019	2020	2021	2022	2023	2024
DWS	336.713	334.638	332.419	330.086	327.658	325.152	322.583

Unit: (100 Million tons).
